# Cannabis Use Variations and Myocardial Infarction: A Systematic Review

**DOI:** 10.3390/jcm13185620

**Published:** 2024-09-22

**Authors:** Jan van Amsterdam, Wim van den Brink

**Affiliations:** 1 Department of Psychiatry, Amsterdam UMC, University of Amsterdam, Meibergdreef 9, P.O. Box 22660, 1100 DD Amsterdam, The Netherlands; w.vandenbrink@amsterdamumc.nl; 2 Research Program Compulsivity, Impulsivity & Attention, Amsterdam Neuroscience, University of Amsterdam, P.O. Box 22660, 1100 DD Amsterdam, The Netherlands

**Keywords:** cannabis, marijuana, myocardial infarction, coronary heart disease, smoking, vaping, edibles, carbon monoxide

## Abstract

**Background:** Cannabis use is associated with an increased risk of coronary heart disease (CHD), including angina pectoris (AP), and myocardial infarction (MI). However, it is not clear whether cannabis use is an independent risk factor of AP and/or MI, because cannabis is often smoked together with tobacco. We investigated whether cannabis is an independent risk factor of MI and whether this risk is similar in cannabis smokers, cannabis vapers, and those who use cannabis edibles. **Methods:** A systematic review was performed, according to the PRISMA guidelines and using Medline (PubMed), Embase, and Google Scholar as databases. **Results:** Twenty-two eligible papers were identified. After adjustment for concurrent tobacco use, cannabis smoking remained significantly associated with incidents of MI, with aORs ranging between 1.03 and 5.24, and particularly high aORs in the younger age group. In never-tobacco smokers, frequent cannabis smoking was also associated with a significant MI risk (aOR = 1.88). Frequent and current cannabis use in any form other than smoking (e.g., vaping, but mostly ingestion) was not associated with a significantly increased cardiovascular risk (frequent use: aOR = 1.00 ns; current use: aOR = 1.31 ns). **Conclusions:** Like tobacco smoking, cannabis smoking may independently provoke MI. Vaping and ingestion of cannabis might be less harmful, probably because absence of combustion prevents exposure to certain toxins in cannabis smoke, including carbon monoxide.

## 1. Introduction

Cannabis is the most widely-used internationally regulated drug in the US, with nearly 22.2 million users each month, 38% of high school students reporting cannabis use [1], and more than half of the adult US population having tried cannabis [2]. In the Behavioural Risk Factor Surveillance System (BRFSS, a yearly telephone survey), the prevalence of last-month cannabis use in the US increased from 10.0% (95% CI: 9.4–10.7%) in 2017 to 13.4% (95% CI: 12.8–12.0%) in 2019 [3]. Smoking cannabis was the most prevalent primary route of administration (76.3%), but cannabis vaping (11.3%) and oral consumption of cannabis (edibles; 12.4% to nearly 30%) are emerging [4,5,6,7]. Especially among youngsters, cannabis vaping and cannabis edibles have become popular. For example, past-month pooled prevalence of adolescent cannabis vaping in the US and Canada has increased 5-fold from 1.6% in 2013–2016 to 8.4% in 2019–2020 for all cannabis users [8,9]. Among US Grade 12 students, 35% and 40% reported vaping and eating cannabis, respectively, in the past 12 months in 2018 [10,11].

Apart from the pulmonary and cancer risk, cannabis smoking is known to be associated with an increased risk of coronary heart disease (CHD), also known as ischemic heart disease, including angina pectoris and myocardial infarction (MI) [12,13,14]. In the 1960s and 1970s, numerous studies documented that tobacco smoking poses a significant risk of CHD, including higher rates of MI and sudden death from CHD, especially in young and middle-aged men and heavy smokers in general [15,16,17,18,19,20]. For instance, a case-controlled study reported a relative risk (RR) of MI of 2.8 (95% CI: 2.0–4.0) for current smokers compared to never-smokers aged 30 to 54 years [20].

In 1975, Aronow & Cassidy suggested that co-use of cannabis and tobacco more often precipitates coronary syndromes (including acute MI) in patients with coronary history compared to those who only smoke tobacco [21]. In 2011, cannabis smoking was added to the list of the potential triggers for MI [22], which was followed by numerous studies showing substantial evidence for an association of frequent cannabis smoking with a higher risk of MI and CHD [23,24,25,26,27]. For instance, an odds ratio of 5.03 (95% CI: 3.5–7.3) was calculated for developing MI after cannabis use in teenagers [28]. The latter study also showed that MI occurs in healthy cannabis users who have a low incidence of cardiac risk factors (e.g., smoking, diabetes).

A wealth of information is available about cannabis-induced MI, but it is not clear whether cannabis use is independently associated with MI. It cannot be excluded that the MI risk of cannabis use is limited to cannabis smoking or that the presumed association is heavily biased by (concurrent) tobacco smoking [29]. We hypothesize that cannabis-related MI is primarily driven by cannabis use in smoked form. Therefore, the aim of the current systematic review is to outline the independent risk of smoked cannabis for CHD, in particular MI. In addition, this review explores the MI risk of non-smoked cannabis, e.g., the risk of vaping or eating cannabis.

## 2. Methods

### 2.1. Search Strategy

A systematic literature review was performed on 20 July 2024, according to the PRISMA guidelines. Utilizing several databases (Medline (PubMed), Embase, and Google Scholar), a comprehensive search was performed for literature concerning the association between cannabis use and myocardial infarction. Keywords, including ‘myocardial infarction’, ‘acute coronary syndrome’, and ‘heart attack’, were employed in addition to various synonyms for cannabis. These keywords were used individually and in combination to select eligible studies. Eligible studies included randomized controlled trials, review articles, meta-analyses, observational cohort studies, observational cross-sectional studies, and case reports referring only to smoking cannabis. Studies in samples with clear co-morbidity for MI, like diabetes, were excluded.

### 2.2. Screening of Records and Data Extraction

**Figure 1:**
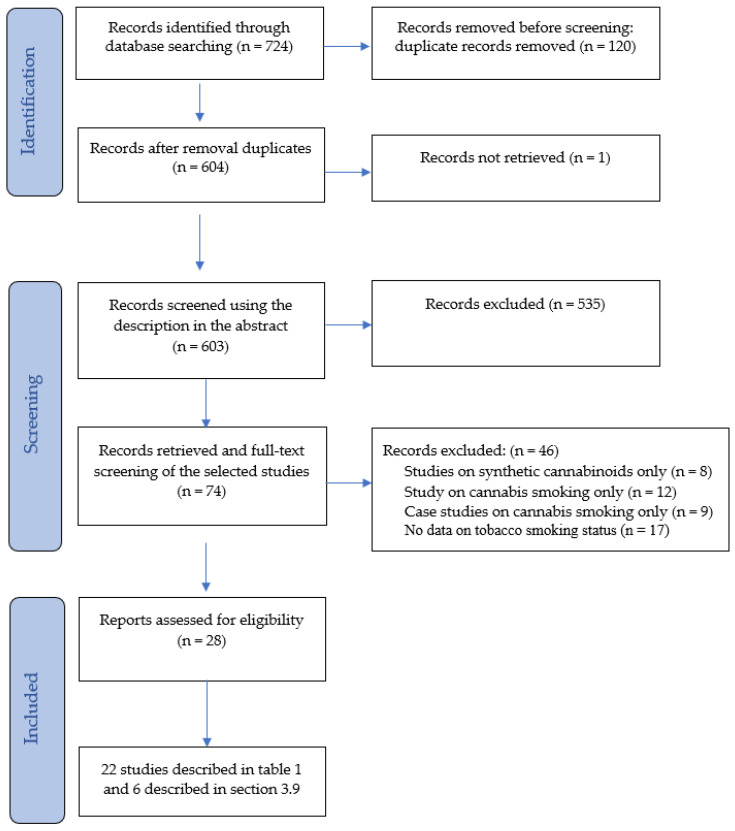
PRISMA flow diagram.

The selection of eligible studies was independently conducted by JvA and WvdB in two rounds. Initially, 724 studies were identified and, after removing duplicates, 604 articles remained. One study could not be retrieved. Of the remaining 603 studies, titles and abstracts were screened to determine eligibility based on the inclusion and exclusion criteria outlined above to identify potentially relevant studies. In a second round, the full texts of 68 studies were comprehensively reviewed to check their eligibility. Finally, 28 studies (including six case reports and one systematic review) were eligible, of which 22 are presented in Table 1. The single case studies on vaping/eating cannabis were selected from the reviews retrieved and briefly described in a separate paragraph (3.9). Figure 1 illustrates the PRISMA flow chart for the identification, screening, and inclusion of the studies. Here, we refer to the ‘Supplement’ for the search string and PRISMA checklist.

### 2.3. Risk of Bias and Quality Assessment

Critical Appraisal Tools (CAT) checklists, developed by the Joanna Briggs Institute (JBI) [47], were used to assess the risk of bias in all included studies. Since this review included studies with different designs, the following appropriate JBI CAT checklists were selected [48]: CAT for observational, analytical cross-sectional studies, CAT for systematic reviews, CAT for observational cohort studies, and CAT for case reports.

The two authors (JvA and WvdB) independently appraised the studies. Any disagreement between them was discussed until a consensus for each score was reached. The items of the JBI CAT checklist were rated as either yes bias (Y), no bias (N), bias unclear (U), or bias not applicable (N/A). As recommended by the JBI manual [47,49], the scoring system and cut-off points to determine high, moderate, or low risk of bias, were decided according to Algarni et al. [50] and agreed by the authors JvA and WvdB, before starting the critical appraisal.

Quality assessment scores for each study were based on the calculated percentage of affirmative responses to the total number of questions. If a criterion was considered not applicable, this point was deducted from the overall score. Studies with a JBI score > 66% were at low risk of bias, scores between 33 and 66% were at moderate risk of bias, and studies with a JBI score less than 33% were deemed at a high risk of bias [50].

## 3. Results

### 3.1. Search Results

Figure 1 shows the PRISMA diagram of the identification, screening, and inclusion of the 28 eligible studies.

### 3.2. Summary of Studies

The articles selected for this review are summarised in Table 1, and detail the sample characteristics, main outcome(s), and literature reference. Studies were classified as (a) studies not adjusting for tobacco use, (b) studies adjusting for tobacco use, (c) studies in non-tobacco users, and (d) studies that included edibles. The total set of 28 eligible studies consists of eighteen cross-sectional investigations [4,23,26,42,43,44,45], three observational cohort studies [21,30,38], one systematic review [46], and six single case studies not mentioned in Table 1.

### 3.3. Risk of Bias

The quality score and assigned risk of bias is represented in Table 2 for all 28 studies. Of the studies, 20 studies were at low risk of bias [4,23,24,32,33,34,36,37,38,39,40,41,43,44,45,51,52,53,54,55], three studies were at moderate risk of bias [21,30,46], and five studies were at high risk of bias [26,31,35,42,56]. Five out of six subjects in the case studies scored low risk of bias and one subject scored a high risk of bias. Tobacco smoking status was, however, unknown in three cases, two cases were smokers, and one case was a non-smoker. The appraisal data for each study is represented in Table S1 of the Supplement.

### 3.4. Studies Not Adjusted for Tobacco Smoking

Numerous reviews have previously outlined the cardiovascular implications of cannabis use [12,58,59,60,61,62,63,64,65,66,67,68,69] (for an overview see [70]). Table 1 summarizes the findings of “studies adjusted for tobacco smoking” included in the current systematic literature search.

Importantly, in a prospective cohort study, Aronow and Cassidy showed, in 10 patients with chronic but stable angina, that smoking one cannabis cigarette significantly decreased the exercise time until angina (by 48–50%) more than either smoking placebo cannabis (8.6%) or smoking one high-nicotine cigarette (23%) [21]. Another important study is the case-cross-over study by Mittleman et al. who examined the self-reported acute effects of cannabis in over 3800 patients and found a 4.8-fold increase in the RR for MI in the first hour after smoking cannabis. In the second hour after smoking, the RR decreased to 1.7 and was no longer significant, suggesting a substantial decrease in cardiac effects in the second hour after cannabis use. The study also found a RR of 3.2 for smoking cannabis in the absence of other potential MI triggers (e.g., regular exertion, cocaine use). Cannabis users were more likely to be current cigarette smokers (68% versus 32%), but the MI risk data were not controlled for combined tobacco-cannabis smoking [32]. Finally, a systemic review of 46 papers also showed in 14 cases that the time from last cannabis use to the onset of MI symptoms is relatively short, i.e., usually within 5 h [71].

In fully adjusted multivariable models, using cross-sectional data from five two-year cycles, between 2009 and 2018, from the National Health and Nutrition Examination Survey representing almost 1000 middle-aged adults, it was shown that, compared to never use, a history of monthly cannabis use was not associated with MI (OR = 0.78;), but the risk of cannabis use in the past month for MI was threefold greater compared to no use within the past month (OR = 2.98) [33].

In summary, abundant evidence is available for up to a 4.8 times higher risk of MI in cannabis smokers with a relatively high risk of an MI within the first two hours after smoking cannabis.

### 3.5. Studies Adjusting for Tobacco Use

The results of the longitudinal, multi-centre CARDIA study, in 5115 young adults in 1985–1986, on the development of CVD over time showed that, after adjustment for tobacco smoking (and many other potential confounders), neither cumulative lifetime nor recent cannabis use were significantly associated with incidents of CVD, transient ischemic attacks or coronary heart disease [34].

A large population-based study using sample patients (2011–2016) with a history of cannabis abuse, including age- and sex-matched cannabis naive controls, investigated the 3-year cumulative incidence of MI as primary outcome. Incidents of MI were significantly higher in the cannabis use group than in controls with a relative risk (RR) of 2.53. After adjustment for a number of confounders, including a greater predilection for tobacco in the cannabis abuse group, cannabis abuse was still significantly associated with incidents of MI (aOR = 1.72) [35].

A retrospective study utilizing the Nationwide Inpatient Sample (NIS) database analysed the trends of MI in young cannabis users aged 18–49 yrs. from 2007 to 2018, i.e., a period in which legalization of cannabis for medical and recreational purposes took place in some states in the US [36]. Cannabis use was reported by 230,497 hospitalized patients (28%). The incidence of acute MI (AMI) among cannabis users consistently increased from 2.36% in 2007 to 6.55% in 2018. Tobacco smoking was identified as an independent risk factor for AMI among cannabis users with an odds ratio (OR) of 2.38, indicating an added risk of MI by tobacco-cannabis co-use. Other results from the NIS showed that the prevalence of coronary artery disease (CAD) was slightly but significantly higher in patients with versus patients without a history of cannabis use (5% vs. 4.6%, *p* < 0.0001); however, the positive associations between cannabis use and rates of CAD were no longer significant after adjustment for a variety of confounders, including co-morbidity and tobacco use [37].

In another large retrospective study, using the NIS database, which included patients with and without a history of cannabis use, hospitalized MI patients showed a small but significant increased risk of MI for those who used cannabis even after adjustment for tobacco smoking (aOR = 1.03; *p* < 0.001) [24].

In a prospective study of HIV-infected men (aged 40–60 years), long-term heavy (daily or weekly) cannabis users compared to cannabis non-users had an increased risk of cardiovascular events, including MI, heart failure, and angina pectoris, after controlling for tobacco use (aOR = 2.5; *p* = 0.016). The risk of cannabis/tobacco co-use for cardiovascular events was additive (OR = 4.8; *p* < 0.01) [38].

Using data from the 2017 BRFSS, a retrospective cross-sectional study was conducted to study the association between cannabis use and self-reported doctor-confirmed cardiovascular disease which included myocardial infarction, angina, coronary heart disease, or a stroke [39]. Remarkably, there was a negative association between cannabis use and cardiovascular disease (OR = 0.65) but statistical significance was lost after adjustment for smoking and other variables [39].

In a retrospective study of hospitalized patients between 2012 and 2014, the risk of MI was compared between the cannabis urine positive group (n = 3638) and the cannabis urine negative group (n = 10,852) [40]. In the >37 yrs. age group, no difference in the risk of MI was observed between the two groups (*p* = 0.48). However, in the 18–36 age group, the risk of MI was considerably higher in the cannabis urine positive group (OR = 2.84). Both tobacco use and cocaine use were found to be significantly higher in cannabis users compared to cannabis non-users. After adjustment for confounding by tobacco smoking and cocaine use, the risk in the 18–36 age cannabis positive group of MI increased almost twofold (OR = 2.84 → OR = 5.24), whereas the risk between the cannabis users and non-users remained the same in the 37–54 age group (OR = 1.11).

Using the data the Third National Health and Examination Survey (NHES), the effect of cannabis on CHD was investigated, using the cardiac infarction and/or injury score (CIIS), in 900 ever-cannabis users of whom 538 were subjects with myocardial injury [41]. Adjusted for potential confounders, including tobacco smoking, ever-cannabis use had 43% increased odds of myocardial injury compared to never users (OR = 1.43) [41].

The results of a National Health and Nutrition Examination Survey (NHANES) study showed a significant OR of ever- versus never-cannabis use for coronary artery disease of 1.90. Similar significant ORs were observed for current cannabis users (OR = 1.98) and heavy cannabis users (OR = 1.99) [42]. These results were consistent in subgroups stratified by tobacco smoking status, amongst other factors.

In summary, adjustment for tobacco smoking often reduced the risk of cannabis use for MI, but, generally, cannabis smoking remained significantly associated with incidents MI with aORs up to 2.84. The risk of cardiovascular events, which includes MI, is particularly high in the younger age group and was reported to be additive in cannabis/tobacco co-use. Tobacco smoking proved to be an independent risk factor for MI among cannabis users.

### 3.6. Studies in Non-Tobacco Users

Using the NIS (2007–2014) database, national trends in hospitalizations for major cardiovascular events, including MI, among young non-tobacco smoking cannabis users were assessed [23]. As compared to non-cannabis users, the rate of hospital admission for MI among 0.7 million (1.3%) current/former cannabis users (no abuse of other substances) was 0.23% compared to 0.14% in non-cannabis users (*p* < 0.001) [23].

The association between cannabis use disorder (CUD) and major adverse cardiac and cerebrovascular events was investigated in an NIS 2019 subsample of older (>65 yrs.) patients without a tobacco use disorder [43]. The prevalence of CUD in this subsample was 0.3% (n = 28,535). Compared to the non-CUD cohort, the CUD cohort reported a higher risk of major adverse cardiac and cerebrovascular events (MACCE: OR 1.20, 95% CI 1.11–1.29, *p* < 0.001). Of the patients with CUD and at risk of cardiovascular disease (CVD), 13.9% reported MACCE. In this group, the CUD subgroup had higher unadjusted rates of MI than the non-CUD group (7.6% vs. 6.0%) [43].

Data from the BRFSS 2016–2020 was used to assess the association of cannabis use in the past 30 days in a general population subsample of never-tobacco smokers (primary cannabis smoking) with self-reported MI (18–74 years). For daily cannabis users in the general population, the aOR for MI was 1.25 (95% CI, 1.07–1.46) and a similar negative effect of daily cannabis use on MI was shown in never-tobacco smokers (aOR = 1.49; 95% CI, 1.03–2.15) [44].

In summary: the rate of MI and hospital admission for MI was higher among non-tobacco smoking current/former cannabis users compared to non-cannabis users. Thus, it seems that cannabis-tobacco co-use does not fully explain the increased cardiovascular risk of cannabis smoking.

### 3.7. Vaping Cannabis

Cigarettes and joints are burned at a temperature of 500–600 °C. In vaping the cannabinoids are heated at around 170 °C to 210 °C and not combusted. Smoking cannabis thus produces significantly higher CO and CO-haemoglobin concentrations 0.25–6 h post-dose compared to vaporization suggesting that vaporized cannabis might be safer than smoked cannabis in terms of its cardiovascular risk. Another advantage of vaporization is that less toxic pyrolytic compounds are produced at vaping temperatures below 200 °C [72]. Except for some case studies [51,52,56], no studies are available on larger cohorts of cannabis vapers and their possible risk of MI, suitable for the current review.

In summary, vaping cannabis is presumably associated with a lower risk of MI, but solid data endorsing this assumption is not available.

### 3.8. Edibles

Apart from some case studies (cf. Table 1), four studies [4,26,45,46] have addressed the risk of MI in a larger sample of users who had orally ingested cannabis or cannabis-like substances as an edible or beverage.

Using pooled data from the 2017 and 2018 cohorts of the BRFSS, Ladha et al. [4] assessed the association between any recent cannabis use and a history of MI among 4610 recent cannabis users. The rates were adjusted for a large variety of confounders, including concomitant tobacco use. A history of MI was significantly associated with smoking cannabis as a primary method of administration (aOR 2.01), but not significantly with vaporizing cannabis (aOR = 2.26) or other forms of cannabis use, including edibles (aOR = 2.36). However, these latter non-significant findings may result from insufficient power due to small samples: cannabis vapers (n = 431), or other non-smoking routes of administration (n = 539), compared to cannabis smokers (n = 3640).

Monte et al. (2019) [45] stratified ED visits between 2014 and 2016 that were at least partially due to cannabis use into 2329 related to cannabis smoking (90.7%) and 238 related to cannabis ingestion (9.3%). ED admissions due to cannabis-related cardiovascular symptoms were significantly more frequent in cannabis ingesting users than in cannabis smokers: 8.0% versus 3.1% of the admissions, with severe adverse cardiovascular events, including myocardial infarction and ventricular dysrhythmia, occurring in both groups (no numbers presented). Many more visits attributable to edible cannabis were, however, due to acute psychiatric symptoms (18.0%) and intoxications (48%).

The pooled 2016–2018 data from the BRFSS was used to evaluate the association between cannabis and reported cardiovascular disease among US adults who never smoked cigarettes [26]. The aOR of frequent cannabis use (all modalities of use, including edibles) for MI or coronary artery disease was 1.88 when compared with non-cannabis users, while the risk for frequent cannabis-only smokers was very similar (aOR = 2.07). Importantly, across all age groups, cannabis use in any form other than smoking (mostly oral ingestion) was not significantly associated with cardiovascular disease: current non-smoking cannabis use (aOR = 1.31) and frequent non-smoking cannabis use (aOR = 1.00) [26]. This shows that cannabis use in any form other than smoking (mostly oral ingestion) was not significantly associated with cardiovascular disease, but the sample was rather small (n = 786) and therefore the study had insufficient power to detect such an association if it existed.

A systematic review and meta-analysis of clinical trials performed with dronabinol (2.5–5 mg) and nabilone (1–3 mg), orally administered on a daily base for 4 to 16 weeks, showed no adverse cardiovascular effects, but again the power to detect such effects were probably too small [46].

### 3.9. Case Reports on Cannabis Edibles and Vaping

A 17-year-old male (tobacco smoking unknown) with no prior medical, cardiac or substance abuse history suffered from chest pain 3–4 h after vaping cannabis. The patient was diagnosed with elevated troponin and ST-segment elevations, pointing to cardiac ischemia [51]. Following cannabis vaping, a 26-year-old male habitual tobacco smoker suffered acute chest pain the next morning. Clinical investigation showed rising troponin levels (from 8.3 to 14.6 ng/L) indicating myocardial infarction [52]. A 70-year-old cannabis naïve male vaped several times, in close succession, a 65% THC concentrate which resulted, within minutes of use, in chest pain, palpitations, and a STEMI progressing to a fatal cardiac arrest [56]. A male tobacco smoker (70-year-old) consumed a cannabis lollipop at a dose of 70 mg (7-fold the recommended dose) which elicited MI [53]. Ingestion of cannabis formulated as a burger by a 55-year-old non-smoking male resulted in elevated troponin I (0.167 ng/mL), chest pain, and NSTEMI [54]. Finally, the ingestion of 600 mg of cannabis, plus an unknown amount of inhaled cannabis, resulted in a 27-year-old male (tobacco smoking unknown) with a high troponin level (peaked > 270,000 pg/mL; normal range: 0–40 pg/mL), STEMI, and a subsequent fatal cerebrovascular accident [55].

**Figure 2:**
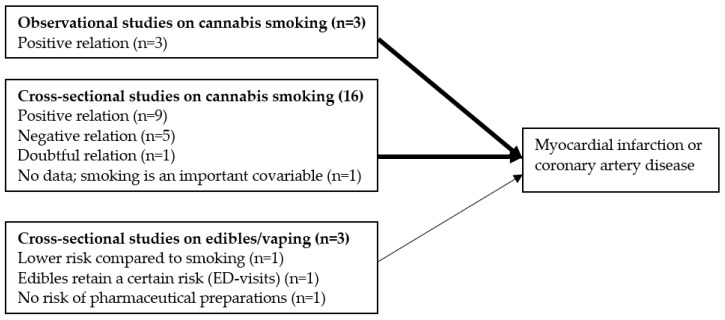
Main findings on the relationship between cannabis use and the risk for MI (number of studies between parenthesis). The thinner arrow reflects the smaller effect size of vaping/edibles compared to smoking cannabis.

Figure 2 summarizes the main findings on the relationship between cannabis use and the risk for MI or coronary artery disease.

## 4. Discussion

This systematic review shows a consistent association of cannabis smoking with an increased risk of MI. Next to tobacco smoking, cannabis smoking seems to retain a certain risk of CHD which may, amongst others, be related to the formation of carbon monoxide (CO) upon smoking. However, this effect is not (fully) explained by the cardiovascular effects of (concurrent) tobacco use. The association between other forms of cannabis use (vaping, edibles) and MI was weaker or non-significant, but this may be partly due to small sample sizes with insufficient power to detect existing effects.

The precise mechanism behind MI provoked by cannabis (and tobacco) smoking is still not fully known, but likely involves carbon monoxide (CO) combined with THC-induced tachycardia and a pro-coagulant effect by polycyclic aromatic hydrocarbons (PHAs) in cannabis smoke, THC or both, akin to the mechanisms observed in tobacco smokers (CO, cardiac stimulation by nicotine and PHA-formation). Overall, blood supply to the heart muscle is insufficient which elicits myocardial ischemia via a mismatch between oxygen supply and demand [73]. Compared to administration of cannabis via vaporization or ingestion, smoking cannabis acutely and dose-dependently increases carbon monoxide (CO) and carboxyhaemoglobin (COHb) concentrations [74,75]. The generation of CO via combustion and subsequent formation of carboxyhaemoglobin in the blood seems to be the main culprit in CHD due to a reduction of oxygen delivery to the myocardium. For example, Aronow and Cassidy (1974) showed that, in patients with chronic but stable angina pectoris, the exercise time to the onset of angina symptoms was decreased by about 48% after smoking a single cannabis cigarette, compared with a decrease of only 9% after smoking a placebo cigarette [30]. Moreover, smoking one cannabis cigarette increased the COHb level significantly more than smoking one high-nicotine cigarette [21]. Finally, the topography of smoking of tobacco and cannabis is quite different: compared to tobacco smoking, cannabis is smoked with 60% larger puff volumes and a 4-fold longer breath-hold time, which may lead to higher exposure levels (internal dose) resulting in a nearly 5-fold increase in COHb levels after cannabis smoking compared to tobacco smoking [76].

The negative impact of tobacco on the MI risk of cannabis smoking may be even more persistent than just described, because smokers are, in addition to CO, repeatedly exposed to a large number of toxic compounds other than CO. These compounds represent, in addition to e.g., lung cancer, a major risk of progressive coronary thrombosis known to be implicated in the pathology of myocardial infarction [77,78]. PHAs found in the tar fraction of both cigarette and cannabis smoke [79] have been shown, at least in experimental models, to accelerate atherosclerosis [80,81]. This pathological element therefore applies to tobacco smoking, as well as cannabis smoking. Moreover, blood platelets are responsive to cannabinoids which explains the pro-coagulant effect of THC [82] an important factor in (coronary) atherosclerosis. Though the data indicate an MI crisis is provoked within 1–2 h of cannabis smoking [32], part of the cannabis-use-related cardiovascular incidents, including atherosclerotic damage and MI, may be due to long-term cannabis use. Such prolonged cannabis use may, like prolonged tobacco smoking, amongst other factors, lead to platelet aggregation, and atherosclerosis progression [83], components known to increase the risk of MI. Collectively, the data show that the precise mechanism of MI induced by both cannabis smoking and tobacco smoking remains to be resolved, including whether THC or other cannabis smoke components have an indirect negative impact on the risk of MI via atherogenesis.

Natural cannabis contains over 60 compounds with varying pharmacological activity [84]. At first glance, it seems, therefore, attractive to include synthetic cannabinoid (SC) use in a review on the MI risk in cannabis users, because SC are single compounds (THC-analogues). However, in contrast to the partial agonist THC (i.e., cannabis) at the CB1 and CB2 receptors, SC are full agonists with a 5–80 times higher potency, which may account for their greater toxicities [85] and the relatively high number of serious incidents, including fatal incidents compared to cannabis [86,87,88]. Another argument to exclude SC from this study is the very low prevalence of SC-use compared to the use of natural cannabis, leading to low numbers of MI cases.

It is not clear yet whether the MI risk in cannabis vapers and people who orally ingest cannabis is really (much) smaller than in cannabis smokers. However, edible cannabis, which avoids exposure to cardiotoxic CO, might be a safer administration route, as suggested by the absence of significant associations with cardiovascular events in some studies. Similarly, vaping leads to virtually no expired CO and is therefore presumably less harmful than smoking cannabis and may offer a safer alternative to smoking, although further research is needed to clarify its cardiovascular effects, especially considering reported cases of MI following vaping (cf. Table 1), although it is not clear whether these observations were biassed by concomitant tobacco smoking or co-morbidities. However, vaping and edibles are popular among younger, healthy men with no cardiovascular risk factors, which may lead to an underestimation of the MI risk. This further implies that it cannot be excluded that the MI patient population using both routes of cannabis administration will change to include older individuals in the future. Indicative of the lower harm of non-smoking forms of cannabis use are the results of a Canadian study from 2012 on cannabis attributable harm showing that, if only non-smoked forms of cannabis were consumed, at least two-thirds of deaths attributable to cannabis use that year would have been avoided [89].

## 5. Conclusions

It is concluded that cannabis use, certainly in smoked form, may have deleterious cardiovascular effects, including provocation of (acute) MI. Therefore, patients with known coronary artery disease should refrain from cannabis use, while those seeking the euphoric effects of THC should consider vaporizers to avoid exposure to the harmful components generated via combustion of either cannabis or tobacco (e.g., when formulated in a joint), including CO [90]. High quality prospective studies are essential to further elucidate the role of cannabis, in particular smoking versus vaporizing and edibles, in precipitating acute coronary syndrome and other cardiovascular problems.

## 6. Limitations

Tobacco smoking is commonly known to be associated with MI. To examine the independent impact of cannabis use on MI, confounding by tobacco smoking must therefore be eliminated. However, tobacco smoking history is often not addressed, nor were data routinely adjusted for concomitant tobacco smoking as a confounder. Unfortunately, tobacco-cannabis co-use among cannabis users is very common (see e.g. [91]), which leads to small sample sizes of non-tobacco smoking cannabis users. The low number also applies to cannabis vapers and those who ingest cannabis (edibles), which may result in null findings (assuming differences between cannabis use modalities exist).

The high variability in the many compounds that natural cannabis contains, including the variation in THC-concentration and the emerging number of cannabis-containing products, may result in large differences in exposure among cannabis users. This variation is not or is poorly addressed in the selected studies and could, therefore, not be studied in this review as separate predictors of myocardial infarction.

Many papers suffer from variability in design and some from poor quality which hinder proper evaluation of the association between cannabis use and MI. Most studies refer to ‘cannabis smoking’ without specifying the form (joint, spliff, or blunt), the frequency of use, and amount of cannabis per session, and the THC-concentration, making it impossible to study the dose-relation between cannabis use in MI. In some studies, angina pectoris was used as the main outcome instead of MI, making results less comparable. In addition, risk factors for MI, like co-morbidities, and use of substances other than cannabis were mostly not addressed or described.

Finally, most of the eligible studies were performed in the US, which may have skewed our results because of major differences in cannabis consumption between the US and Europe. This could be considered a limitation. Although we used PubMed, Embase, and Google Scholar, which account for 93% of published literature on a given search [92], we were not able to retrieve more eligible studies from either Europe or Australia.

## Figures and Tables

**Table 1:** Studies on cannabis-related MI. Outcome was either adjusted for concomitant tobacco use, tobacco smokers were compared with non-tobacco smokers, or the study was performed on never-tobacco smokers.

Reference	Design	Sample Characteristics	Main Outcome	Comment on Association *
Studies not adjusting for tobacco use				
(Aronow & Cassidy, 1974) [30]	Prospective cohort	Patients with chronic but stable angina (n = 10)	Exercise time to onset of angina symptoms: decrease of 48% and 8.6% after smoking one cannabis cigarette and one cannabis placebo cigarette, respectively.	Positive
(Aronow & Cassidy, 1975) [21]	Prospective cohort	Patients with chronic but stable angina (n = 10)	Exercise time to onset of angina symptoms: decrease of 50% and 23% after smoking one cannabis cigarette and one high-nicotine cigarette, respectively.	Positive
(Jouanjus et al., 2014) [31]	Retrospective study	Hospitalised ACS cases * (n = 20)	55% of hospitalised ACS cases related to cannabis use were concomitant tobacco smokers; smoking status not declared in nine cases.	Doubtful
(Mittleman et al., 2001) [32]	Case-cross-over study	N ≥ 3800 patients	4.8-fold (95% CI: 2.9–9.5) increase in the RR for self-reported MI in the first hour after smoking cannabis; in the second hour after smoking, the RR decreased to 1.7 (95% CI: 0.6–5.1).	Positive
(Corroon et al., 2023) [33]	Cross-sectional survey (2009–2018)	N = 9769; 35–59 yrs. old	OR of cannabis use in the past month for MI = 2.98 (95% CI: 1.08–8.60) compared to no use.	Positive
Studies adjusting for tobacco use				
(Reis et al., 2017) [34]	CARDIA longitudinal, multi-centre study	N = 5115 young adults (aged 18 to 30 yrs.); 84% reporting ever cannabis use	In tobacco-smoking-adjusted analyses, cumulative lifetime and recent cannabis use was not associated with incident CVD, transient ischemic attacks or coronary heart disease.	Negative
(Chami & Kim, 2019) [35]	Multicentre study (2011–2016)	Patients with a history of cannabis use (n = 292,770; 37.4 ± 15 yrs. old)	3-year cumulative incidence of MI was higher in cannabis users vs. controls (RR = 2.53; 95% CI, 2.45–2.61). Following adjustment for confounders, including tobacco smoking, the aOR was 1.72 (95% CI: 1.67–1.77).	Positive
(Sandhyavenu et al., 2023) [36]	Retrospective NIS study	Cannabis using hospitalized MI patients (n = 230,497; 18–49 yrs. old)	Tobacco smoking was an independent risk factor for MI among cannabis users (OR: 2.38, 95% CI: 2.23–2.54).	Smoking is a significant covariable
(Kalla et al., 2018) [37]	Retrospective study NIS study (2009–2010)	Cannabis users (n = 316,397, mean age: 33.1 yrs. old)	Prevalence of CADS was higher in cannabis users (n = 397) compared to non-users (5% vs. 4.6%, *p* < 0.0001), but no difference after adjustment for tobacco use.	Negative
(Desai et al., 2017) [24]	Retrospective NIS study	Hospitalized cannabis using MI patients (n = 35,771; 49.3 ± 10.7 yrs. old)	Compared to patients without a history of cannabis use, aOR of cannabis use for MI adjusted for tobacco use was 1.03 (95% CI: 1.018–1.045).	Positive
(Lorenz et al., 2017) [38]	Prospective study	HIV-infected men (n = 558)	Compared to nonusers and adjusted for tobacco use, daily or weekly cannabis use: OR = 2.51; 95% CI: 1.18–5.31; daily or weekly co-use of cannabis and tobacco: OR = 4.8; 95% CI: 1.04–4.51.	Positive
(Jivanji et al., 2020) [39]	Retrospective study of 2017 BRFSS	N = 56,742; cannabis users: n = 2989 < 65 yrs. old and n = 409 ≥ 65 yrs. old	OR of cannabis use for cardiovascular disease was 0.65 (95% CI: 0.50–0.84), but non-significant after adjustment for smoking and other variables (OR = 0.74; 95% CI: 0.54–1.01).	Negative
(Karki et al., 2022) [40]	Retrospective study	Hospitalized patients (n = 3638 cannabis+: and n = 10,852 cannabis−)	Cannabis use and risk of MI: <54 years of age: no difference (*p* = 0.48); in those aged 18–36 yrs.: cannabis+ vs. cannabis−: OR = 2.84 (95% CI: 1.14–7.07); aOR after adjustment for tobacco use and cocaine use was 5.24 (95% CI: 1.84–16.93).	Positive
(Skipina et al., 2021) [41]	Retrospective study of NHES survey	900 ever-cannabis users (26% of total sample; 538 with myocardial injury)	Adjusted for tobacco smoking, aOR of the risk of ever-cannabis use for myocardial injury (CIIS ≥ 10) was 1.43 (95% CI: 1.14–1.80).	Positive
(Skipina et al., 2022) [42]	Retrospective study of NHANES survey	N = 12,543 participants (53% self-reported ever cannabis use; 39.3 ± 11.6 yrs. old)	OR of cannabis ever used (versus never) for physician diagnosed CAD was 1.90 (95% CI: 1.24–2.93); current cannabis use (OR = 1.98; 95% CI: 1.11–3.54); and heavy cannabis use (OR = 1.99; 95% CI: 1.02–3.89). Same results in tobacco smoking stratified groups.	Positive
Studies in non-tobacco users				
(Desai et al., 2019) [23]	Retrospective study of NIS 2007–2014	Non-tobacco smoking cannabis using MI patients (18–39 yrs. old)	Rate of hospital admission for MI is lower in cannabis users compared to non-cannabis users (0.14% vs. 0.23%; *p* < 0.001).	Negative
(Mondal et al., 2024) [43]	Retrospective study of NIS 2009	Non-tobacco smoking patients with CUD (n = 28,535)	In non-tobacco smoking patients with higher CUD unadjusted rate of MI (7.6% vs. 6%) compared to non-CUD.	Positive
(Jeffers et al., 2024) [44]	Cross-sectional study of 2016 to 2020 BRFSS data	Daily cannabis users (n = 12,331 tobacco smokers and 2892 never-tobacco smokers)	Self-reported MI in cannabis smokers: aOR = 1.25 (95% CI, 1.07–1.46); in never-tobacco smokers: aOR = 1.49; 95% CI: 0.93–2.38.	Smoking is a significant covariable
(Shah et al., 2021) [26]	Cross-sectional study of 2016 to 2018 BRFSS data	Cannabis users who have reported MI or CAD (n = 133,706; 18–74 yrs. old)	aOR of cannabis use for reported MI or CAD: all frequent use modalities, incl. edibles: aOR = 1.88 (95% CI: 1.15–3.08) compared to non-use. Frequent smoking only: aOR = 2.07 (95% CI: 1.21–3.56); frequent use in any form other than smoking (mostly ingestion): aOR = 1.00 (95% CI: 0.44–2.31).	Positive
Studies that included edibles				
(Ladha et al., 2021) [4]	Cross-sectional study using BRFSS 2017 to 2018 data	Cannabis users with a history of MI (n = 4610)	History of MI: Cannabis smokers ^#^: aOR = 2.01, 95% CI: 1.02–3.98; Vapers: aOR = 2.26, 95% CI: 0.58–8.82 (N.S.); and Other forms including edibles: aOR = 2.36, 95% CI: 0.81–6.88 (N.S.).	Higher risk for smoking compared to edibles and vaping
(Monte et al., 2019) [45]	Retrospective study	Cannabis use related ED-visits (n = 2567)	ED visits attributable to edible cannabis were more likely due to cardiovascular symptoms (8.0% vs. 3.1%; *p* < 0.001).	Edibles retain a risk of MI;
(Bajtel et al., 2022) [46]	Systematic review	Placebo-controlled clinical studies	In 16 trials (n = 903 patients) no adverse cardiovascular effects following dronabinol (2.5–5 mg p.o.) or nabilone (1–3 mg p.o.), daily for several 4–16 wks.	No risk of edibles for MI

* ACS: acute coronary syndrome; MI: myocardial infarction; NIS: National Inpatient Sample; RR: relative risk; BRFSS: Behavioral Risk Factor Surveillance System; CUD: cannabis use disorder; ED: Emergency Department; CAD: coronary artery disease; CIIS: cardiac infarction and/or injury score ^#^ smoking cannabis as the primary method of consumption.

**Table 2:** Quality score and risk of bias in the eligible studies using the JBI Critical Appraisal Tool.

Authors, Year	Ref.	Type of Study	Quality Score	Risk of Bias
Aronow & Cassidy, 1974	[30]	Observational, cohort study	36%	Moderate
Aronow & Cassidy, 1975	[21]	Observational, cohort study	36%	Moderate
Jouanjus et al., 2014	[31]	Observational, analytical cross-sectional	12%	High
Mittleman et al., 2001	[32]	Observational, analytical cross-sectional	75%	Low
Corroon et al., 2023	[33]	Observational, analytical cross-sectional	88%	Low
Reis et al., 2017	[34]	Observational, analytical cross-sectional	88%	Low
Chami & Kim, 2019	[35]	Observational, analytical cross-sectional	25%	High
Sandhyavenu et al., 2023	[36]	Observational, analytical cross-sectional	88%	Low
Kalla et al., 2018	[37]	Observational, analytical cross-sectional	88%	Low
Desai et al., 2017	[24]	Observational, analytical cross-sectional	88%	Low
Lorenz et al., 2017	[38]	Observational, cohort study	91%	Low
Jivanji et al., 2020	[39]	Observational, analytical cross-sectional	88%	Low
Karki et al., 2022	[40]	Observational, analytical cross-sectional	88%	Low
Skipina et al., 2021	[41]	Observational, analytical cross-sectional	88%	Low
Skipina et al., 2022	[42]	Observational, analytical cross-sectional	12%	High
Desai et al., 2019	[23]	Observational, analytical cross-sectional	75%	Low
Mondal et al., 2024	[43]	Observational, analytical cross-sectional	88%	Low
Jeffers et al., 2024	[44]	Observational, analytical cross-sectional	88%	Low
Shah et al., 2021	[26]	Observational, analytical cross-sectional	25%	High
Ladha et al., 2021	[4]	Observational, analytical cross-sectional	88%	Low
Monte et al., 2019	[45]	Observational, analytical cross-sectional	88%	Low
Bajtel et al., 2022	[46]	Systematic review	45%	Moderate
Case reports on vaping/edibles				
Schreier et al., 2020	[51]	Case study	75%	Low
Hendrickson et al., 2020	[56]	Case study	12%	High
Rahman & Alqaisi, 2023	[52]	Case study	75%	Low
Saunders & Stevenson, 2019	[53]	Case study	75%	Low
Tirkey & Gupta, 2016	[57]	Case study	Could not be retrieved	-
Kariyanna et al., 2020	[54]	Case study	75%	Low
Lavertue et al., 2023	[55]	Case study	63%	Low

JBI: Joanna Briggs Institute; Ref.: reference number.

## Supplementary Materials

The following supporting information can be downloaded at: https://www.mdpi.com/article/10.3390/jcm13185620/s1, Search string used; Table S1: Appraisal according to Critical Appraisal Tools (CAT) checklists developed by the Joanna Briggs Institute (JBI); Table S2: PRISMA 2020 Checklist.
