# Supplementary material for: Cannabis Use Variations and Myocardial Infarction: A Systematic Review
Source: J Clin Med. 2024 Sep 22;13(18):5620. doi: 10.3390/jcm13185620 (PMC11433637; doi:10.3390/jcm13185620)
Supplement: Supplementary file 1 [file jcm-13-05620-s001.zip › jcm-3181889-supplementary.pdf]

Supplement

Search string used to retrieve relevant data from databases (Medline (PubMed), Embase, and Google Scholar):

("cannabis"[All Fields] OR "bhang"[All Fields] OR "marijuana"[All Fields] OR "marihuana"[All Fields] OR "thc"[All Fields]) AND ("acute coronary syndrome"[All Fields] OR "myocardial infarction"[All Fields] OR "heart attack"[All Fields] OR "coronary heart disease"[All Fields] OR "coronary artery disease"[All Fields] OR "myocard infarct"[All Fields] OR "Myocardial Infarction"[Title] OR "chest pain"[All Fields] OR "cardiac arrest"[All Fields] OR "angina"[All Fields] OR "acute ischemic stroke" OR "troponin"[All Fields])

Table S1. Appraisal according to Critical Appraisal Tools (CAT) checklists developed by the Joanna Briggs Institute (JBI).

**Study**	**REF**	**Q1**	**Q2**	**Q3**	**Q4**	**Q5**	**Q6**	**Q7**	**Q8**	**Q9**	**Q10**	**Q11**	**Total score**
**Cohort Studies**[1]													
Aronow & Cassidy, 1974	48	N/A	N/A	Y	N	N	Y	Y	N/A	N/A	N/A	Y	4/11
Aronow & Cassidy, 1975	21	N/A	N/A	Y	N	N	Y	Y	N/A	N/A	N/A	Y	4/11
Lorenz et al., 2017	49	N	Y	Y	Y	Y	Y	Y	Y	Y	Y	Y	10/11
**Systematic reviews**[2]													
Bajtel et al., 2022	56	N	Y	Y	Y	Y	Y	UC	Y	Y	UC	UC	5/11
**Cross Sectional studies**[3]													
Jouanjus et al., 2014	34	N	N	Y	UC	UC	N	N	N	-	-	-	1/8
Mittleman et al., 2001	35	N	Y	Y	Y	Y	N	Y	Y	-	-	-	6/8

Corroon et al., 2023	36	N	Y	Y	Y	Y	Y	Y	Y	-	-	-	7/8
Reis et al., 2017	37	N	Y	Y	Y	Y	Y	Y	Y	-	-	-	7/8
Chami & Kim, 2019	38	N	Y	Y	N	N	N	Y	N	-	-	-	2/8
Sandhyavenu et al., 2023	39	N	Y	Y	Y	Y	Y	Y	Y	-	-	-	7/8
Kalla et al., 2018	40	N	Y	Y	Y	Y	Y	Y	Y	-	-	-	7/8
Desai et al., 2017	24	N	Y	Y	Y	Y	Y	Y	Y	-	-	-	7/8
Jivanji et al., 2020	41	N	Y	Y	Y	Y	Y	Y	Y	-	-	-	7/8
Karki et al., 2022	42	N	Y	Y	Y	Y	Y	Y	Y	-	-	-	7/8
Skipina et al., 2021	43	N	Y	Y	Y	Y	Y	Y	Y	-	-	-	7/8
Skipina et al., 2022	44	N	N	Y	UC	UC	N	N	N	-	-	-	1/8
Desai et al., 2019	23	N	Y	Y	Y	Y	N	Y	Y	-	-	-	6/8
Mondal et al., 2024	45	N	Y	Y	Y	Y	Y	Y	Y	-	-	-	7/8
Jeffers et al., 2024	46	N	Y	Y	Y	Y	Y	Y	Y	-	-	-	7/8
Shah et al., 2021	26	N	Y	Y	N	N	N	Y	N	-	-	-	2/8
Ladha et al., 2021	4	N	Y	Y	Y	Y	Y	Y	Y	-	-	-	7/8
Monte et al., 2019	47	N	Y	Y	Y	Y	Y	Y	Y	-	-	-	7/8
**Case studies on vaping or edibles[4]**													
Schreier et al.,	50	Y	N	Y	Y	Y	Y	Y	N	-	-	-	6/8

2020													
Hendrickson et al., 2020	51	N	N	N	N	N	Y	N	N	-	-	-	1/8
Rahman & Alqaisi, 2023	52	Y	N	Y	Y	Y	Y	Y	N	-	-	-	6/8
Saunders & Stevenson, 2019	53	Y	N	Y	Y	Y	Y	Y	N	-	-	-	6/8
Kariyanna et al., 2020	54	Y	N	Y	Y	Y	Y	Y	N	-	-	-	6/8
Lavertue et al., 2023	55	Y	N	UC	Y	Y	Y	Y	N	-	-	-	5/8

N: no bias; Y: yes bias; UC: bias unclear; N/A: bias not applicable; REF: reference number.

[1]JBI checklist questions for cohort studies:
1) Were the two groups similar and recruited from the same population?
2) Were the exposures measured similarly to assign people to both exposed and unexposed groups?
3) Was the exposure measured in a valid and reliable way?
4) Were confounding factors identified?
5) Were strategies to deal with confounding factors stated?
6) Were the groups/participants free of the outcome at the start of the study (or at the moment of exposure)?
7) Were the outcomes measured in a valid and reliable way?
8) Was the follow up time reported and sufficient to be long enough for outcomes to occur?
9) Was follow up complete, and if not, were the reasons to loss to follow up described and explored?
10) Were strategies to address incomplete follow up utilized?
11) Was appropriate statistical analysis used?

[2]JBI checklist questions for systematic reviews and research syntheses:
1) Is the review question clearly and explicitly stated?
2) Were the inclusion criteria appropriate for the review question?
3) Was the search strategy appropriate?
4) Were the sources and resources used to search for studies adequate?
5) Were the criteria for appraising studies appropriate?
6) Was critical appraisal conducted by two or more reviewers independently?
7) Were there methods to minimize errors in data extraction?
8) Were the methods used to combine studies appropriate?
9) Was the likelihood of publication bias assessed?
10) Were recommendations for policy and/or practice supported by the reported data?
11) Were the specific directives for new research appropriate?

[3]JBI checklist questions for analytical cross-sectional studies:
1) Were the criteria for inclusion in the sample clearly defined?
2) Were the study subjects and the setting described in detail?
3) Was the exposure measured in a valid and reliable way?

4) Were objective, standard criteria used for measurement of the condition?
5) Were confounding factors identified?
6) Were strategies to deal with confounding factors stated?
7) Were the outcomes measured in a valid and reliable way?
8) Was appropriate statistical analysis used?

[4]JBI checklist questions for case series:
1) Were patient's demographic characteristics clearly described?
2) Was the patient's history clearly described and presented as a timeline?
3)Was the current clinical condition of the patient on presentation clearly described?
4) Were diagnostic tests or assessment methods and the results clearly described?
5) Was the intervention(s) or treatment procedure(s) clearly described?
6) Was the post-intervention clinical condition clearly described?
7) Were adverse events (harms) or unanticipated events identified and described?
8) Does the case report provide takeaway lessons?

Table S2. **PRISMA 2020 Checklist**

Section and Topic	Item #	Checklist item	Location where item is reported
**TITLE**			
Title	1	Identify the report as a systematic review.	Cf. title and Methods section
**ABSTRACT**			
Abstract	2	See the PRISMA 2020 for Abstracts checklist.	2
**INTRODUCTION**			
Rationale	3	Describe the rationale for the review in the context of existing knowledge.	Cf Introduction
Objectives	4	Provide an explicit statement of the objective(s) or question(s) the review addresses.	Cf Introduction and Methods
**METHODS**			
Eligibility criteria	5	Specify the inclusion and exclusion criteria for the review and how studies were grouped for the syntheses.	Methods section
Information sources	6	Specify all databases, registers, websites, organisations, reference lists and other sources searched or consulted to identify studies. Specify the date when each source was last searched or consulted.	Methods section
Search strategy	7	Present the full search strategies for all databases, registers and websites, including any filters and limits used.	Methods section and Supplement
Selection process	8	Specify the methods used to decide whether a study met the inclusion criteria of the review, including how many reviewers screened each record and each report retrieved, whether they worked independently, and if applicable, details of automation tools used in the process.	Methods section
Data collection process	9	Specify the methods used to collect data from reports, including how many reviewers collected data from each report, whether they worked independently, any processes for obtaining or confirming data from study investigators, and if applicable, details of automation tools used in the process.	Methods section
Data items	10a	List and define all outcomes for which data were sought. Specify whether all results that were compatible with each outcome domain in each study were sought (e.g. for all measures, time points, analyses), and if not, the methods used to decide which results to collect.	Methods section
	10b	List and define all other variables for which data were sought (e.g. participant and intervention characteristics, funding sources). Describe any assumptions made about any missing or unclear information.	Methods section
Study risk of bias	11	Specify the methods used to assess risk of bias in the included studies, including details of the tool(s) used, how many reviewers assessed	Methods

Section and Topic	Item #	Checklist item	Location where item is reported
assessment		each study and whether they worked independently, and if applicable, details of automation tools used in the process.	section
Effect measures	12	Specify for each outcome the effect measure(s) (e.g. risk ratio, mean difference) used in the synthesis or presentation of results.	N/A
Synthesis methods	13a	Describe the processes used to decide which studies were eligible for each synthesis (e.g. tabulating the study intervention characteristics and comparing against the planned groups for each synthesis (item #5)).	N/A
	13b	Describe any methods required to prepare the data for presentation or synthesis, such as handling of missing summary statistics, or data conversions.	N/A
	13c	Describe any methods used to tabulate or visually display results of individual studies and syntheses.	N/A
	13d	Describe any methods used to synthesize results and provide a rationale for the choice(s). If meta-analysis was performed, describe the model(s), method(s) to identify the presence and extent of statistical heterogeneity, and software package(s) used.	N/A
	13e	Describe any methods used to explore possible causes of heterogeneity among study results (e.g. subgroup analysis, meta-regression).	N/A
	13f	Describe any sensitivity analyses conducted to assess robustness of the synthesized results.	N/A
Reporting bias assessment	14	Describe any methods used to assess risk of bias due to missing results in a synthesis (arising from reporting biases).	Methods section
Certainty assessment	15	Describe any methods used to assess certainty (or confidence) in the body of evidence for an outcome.	N/A
**RESULTS**			
Study selection	16a	Describe the results of the search and selection process, from the number of records identified in the search to the number of studies included in the review, ideally using a flow diagram.	Fig. 1
	16b	Cite studies that might appear to meet the inclusion criteria, but which were excluded, and explain why they were excluded.	Fig. 1
Study characteristics	17	Cite each included study and present its characteristics.	Table 1
Risk of bias in studies	18	Present assessments of risk of bias for each included study.	Table 2 and S1 Table 1
Results of individual studies	19	For all outcomes, present, for each study: (a) summary statistics for each group (where appropriate) and (b) an effect estimate and its precision (e.g. confidence/credible interval), ideally using structured tables or plots.	Table 1
Results of syntheses	20a	For each synthesis, briefly summarise the characteristics and risk of bias among contributing studies.	N/A
	20b	Present results of all statistical syntheses conducted. If meta-analysis was done, present for each the summary estimate and its precision (e.g. confidence/credible interval) and measures of statistical heterogeneity. If comparing groups, describe the direction of the effect.	N/A
	20c	Present results of all investigations of possible causes of heterogeneity among study results.	N/A
	20d	Present results of all sensitivity analyses conducted to assess the robustness of the synthesized results.	N/A
Reporting biases	21	Present assessments of risk of bias due to missing results (arising from reporting biases) for each synthesis assessed.	N/A
Certainty of evidence	22	Present assessments of certainty (or confidence) in the body of evidence for each outcome assessed.	Cf. Limitations

Section and Topic	Item #	Checklist item	Location where item is reported
**DISCUSSION**			
Discussion	23a	Provide a general interpretation of the results in the context of other evidence.	Cf Discussion
	23b	Discuss any limitations of the evidence included in the review.	Cf. Limitations
	23c	Discuss any limitations of the review processes used.	Cf. Limitations
	23d	Discuss implications of the results for practice, policy, and future research.	Conclusion
**OTHER INFORMATION**			
Registration and protocol	24a	Provide registration information for the review, including register name and registration number, or state that the review was not registered.	Pending
	24b	Indicate where the review protocol can be accessed, or state that a protocol was not prepared.	Not prepared
	24c	Describe and explain any amendments to information provided at registration or in the protocol.	N/A
Support	25	Describe sources of financial or non-financial support for the review, and the role of the funders or sponsors in the review.	No support
Competing interests	26	Declare any competing interests of review authors.	No competing interests
Availability of data, code and other materials	27	Report which of the following are publicly available and where they can be found: template data collection forms; data extracted from included studies; data used for all analyses; analytic code; any other materials used in the review.	No other data generated
